# Progressive metastatic pheochromocytoma induced by multiple endocrine neoplasia type 2A with a lethal outcome

**DOI:** 10.1002/iju5.12514

**Published:** 2022-09-06

**Authors:** Koshiro Nishimoto, Noriaki Lukas Santo, Masato Yonamine, Kazuhiro Takekoshi, Go Kaneko, Suguru Shirotake, Hisayo Fukushima, Yoshitaka Okada, Masanori Yasuda, Akihiro Sakurai, Masafumi Oyama, Kento Kanao

**Affiliations:** ^1^ Department of Uro‐Oncology Saitama Medical University International Medical Center Hidaka Japan; ^2^ Laboratory of Laboratory/Sports Medicine, Division of Clinical Medicine, Faculty of Medicine University of Tsukuba Tsukuba Japan; ^3^ Department of Cancer Genomic Medicine Saitama Medical University International Medical Center Hidaka Japan; ^4^ Department of Diagnostic Radiology Saitama Medical University International Medical Center Hidaka Japan; ^5^ Department of Diagnostic Pathology the Saitama Medical University International Medical Center Hidaka Japan; ^6^ Department of Medical Genetics and Genomics Sapporo Medical University School of Medicine Sapporo Japan

**Keywords:** chemotherapy, embolization, Metastatic pheochromocytoma, multiple endocrine neoplasia type 2, thrombosis

## Abstract

**Introduction:**

Patients with multiple endocrine neoplasia type 2A (MEN2A) harboring a pathological variant in the RET gene are characterized by medullary thyroid carcinoma (MTC), pheochromocytoma, and hyperparathyroidism. Although pheochromocytoma is currently defined as a malignant tumor, MEN2A‐associated pheochromocytoma is known to have a small risk of metastasis.

**Case presentation:**

The case was a 62‐year‐old Japanese male with bilateral pheochromocytoma, multiple metastases in the liver and bones, and a cardiac thrombus. Genetic testing revealed a pathological variant at codon 634 of the RET gene, thereby leading a diagnosis of MTC. We considered that the multiple metastases were due to MTC; however, a liver biopsy revealed metastasis of pheochromocytoma.

**Conclusion:**

When pheochromocytoma precedes MTC, the diagnosis of MEN2A may be difficult.

**Abbreviations & Acronyms**
MEN2A: endocrine neoplasia type 2A
MTC: medullary thyroid carcinoma
PPGL: metaiodobenzylguanidine
RET: pheochromocytoma/paraganglioma
MIBG: Ret proto‐oncogene

**Keynote message**

We encountered severely metastasized pheochromocytoma in a patient with multiple endocrine neoplasia type 2A. Multiple metastases were attributed to pheochromocytoma rather than medullary thyroid carcinoma. Genetic testing and serum calcitonin measurement provided invaluable information on the patient.

## Introduction

All pheochromocytoma/paraganglioma (PPGL) have the potential to metastasize to non‐chromaffin tissues; therefore, the prefix term “benign” was abandoned in the 2017 World Health Organization classification of endocrine tumors.^1^ Our study group recently reported the prevalence of germline variants in Japanese patients with PPGL,^2^ in which 32.4% of patients harbored germline variants in seven genes including the Ret proto‐oncogene (RET). Some variants in RET cause multiple endocrine neoplasia type 2A (MEN2A),^3^, ^4^ which is characterized by medullary thyroid carcinoma (MTC, penetration rate: 100%), pheochromocytoma (approximately 50%), and hyperparathyroidism (approximately 10%).^5^ Although pheochromocytoma is defined as malignant, cases with actual malignant potential are rare in MEN2A (<5%).^6^ We herein report an elder case with MEN2A, which was diagnosed followed by severely metastatic pheochromocytoma.

## Case report

**Fig. 1:**
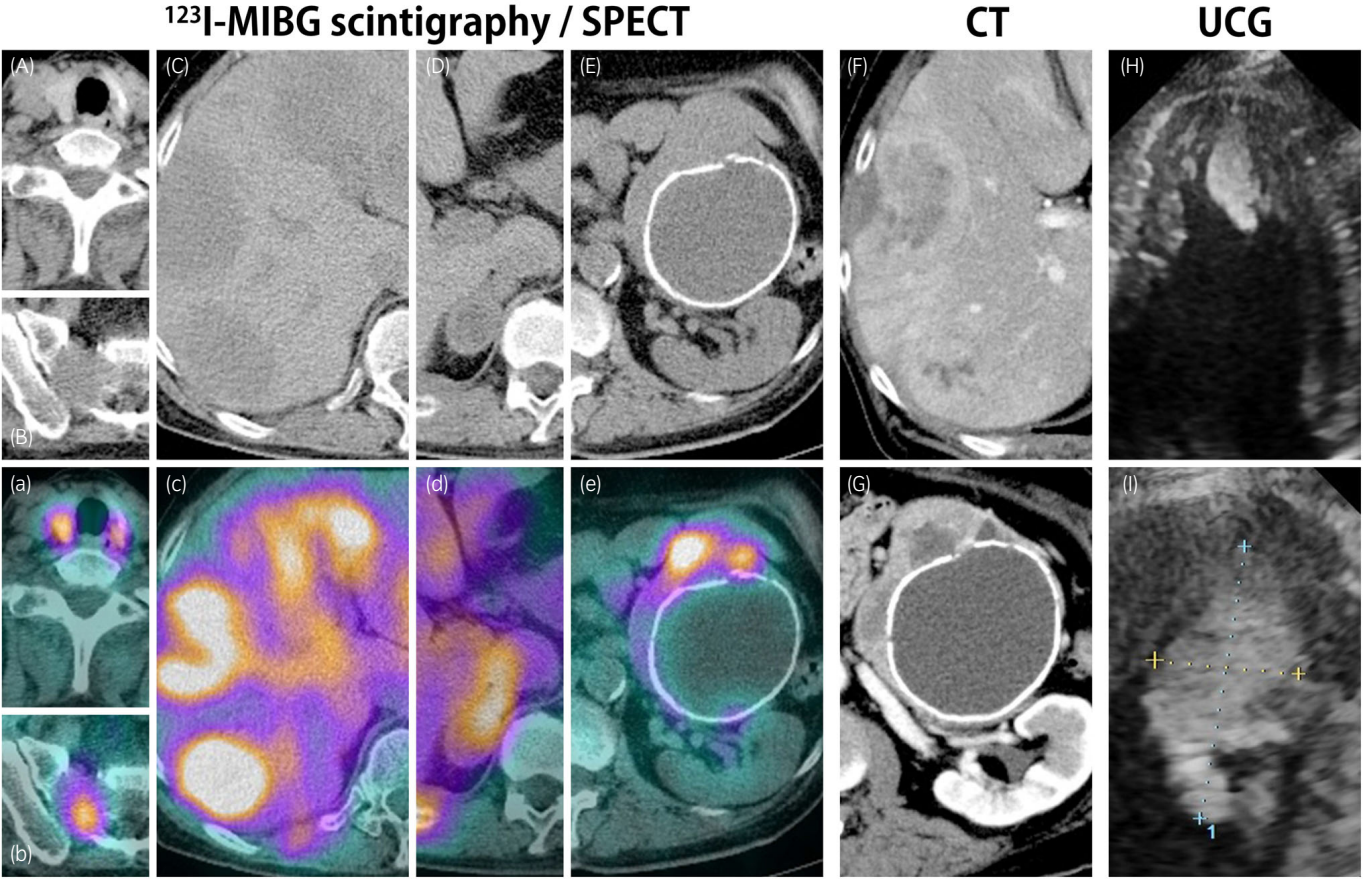
Imaging study of the present case, SIMC‐Uro #11,956*. CT without contrast (A‐E) and ^123^I‐metaidobenzylguanidine (MIBG) scintigraphy (a‐e) and CT with contrast (F and G) on admission, showing the multiple accumulations of MIBG in the thyroid, liver, bones, bilateral adrenal glands, and sacrum, and the left adrenal gland with a size of 120 mm accompanied by internal calcification and a cystic lesion. Cardiac ultrasonography (H and I) showing a 28‐mm hyperechoic embolus at the apex of the heart. *: A unique non‐sequential patient control number in the Department of Uro‐oncology, Saitama Medical University International Medical Center.

A 62‐year‐old Japanese male complained of severe back pain. He had been using candesartan and amlodipine for moderate hypertension. CT revealed bilateral adrenal tumors with multiple metastases in the bones and liver. ^123^I‐metaiodobenzylguanidine (MIBG) scintigraphy and CT showed the accumulations of MIBG in the thyroid (Fig. 1A, a) and tumors with MIBG accumulation in bones (Fig. 1B, b), liver (Fig. 1C, c), and bilateral adrenal glands (Fig. 1D‐E, d‐e). Cardiac ultrasonography incidentally revealed a 28‐mm hyperechoic lesion in the left ventricle (Fig. 1H‐I). Thus, he was diagnosed as metastatic pheochromocytoma.

**Fig. 2:**
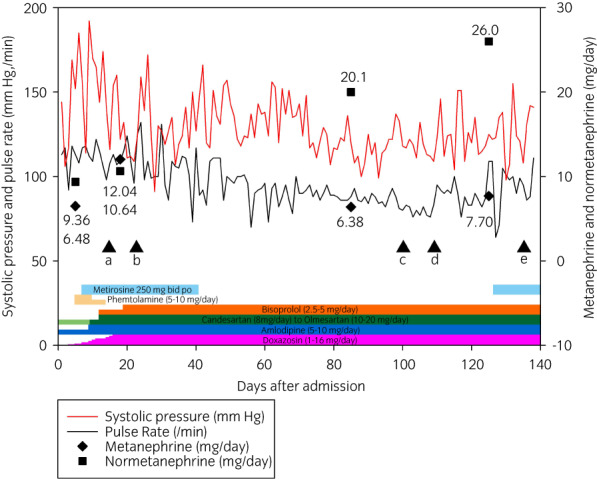
The clinical course of the present case. (a) CVD first course Day 1, (b) left leg amputation, (c) CT‐guided biopsy of the liver, (d) thyroidectomy, (e) trans‐arterial embolization of the liver.

The patient initially received palliative radiotherapy but was urgently admitted to the intensive care unit for the exacerbation of pain (Day 11). He exhibited significant sweating, a burning sensation, and headache due to the hypertensive crisis (Fig. 2). Plasma levels of adrenaline and noradrenaline were very high (1,706 [normal range: 0–80] pg/mL and 10,792 [90–240] pg/mL, respectively). Urinary catecholamine excretion levels of metanephrine and normetanephrine were markedly high (12.0 [0.04–0.20] mg/day and 10.6 [0.09–0.28] mg/day, respectively). Doxazosin and intravenous phentolamine partially attenuated the crisis. An additional treatment with metyrosine (500 mg/day), approved for use in clinical settings in Japan since 2019, markedly ameliorated the crisis.

**Fig. 3:**
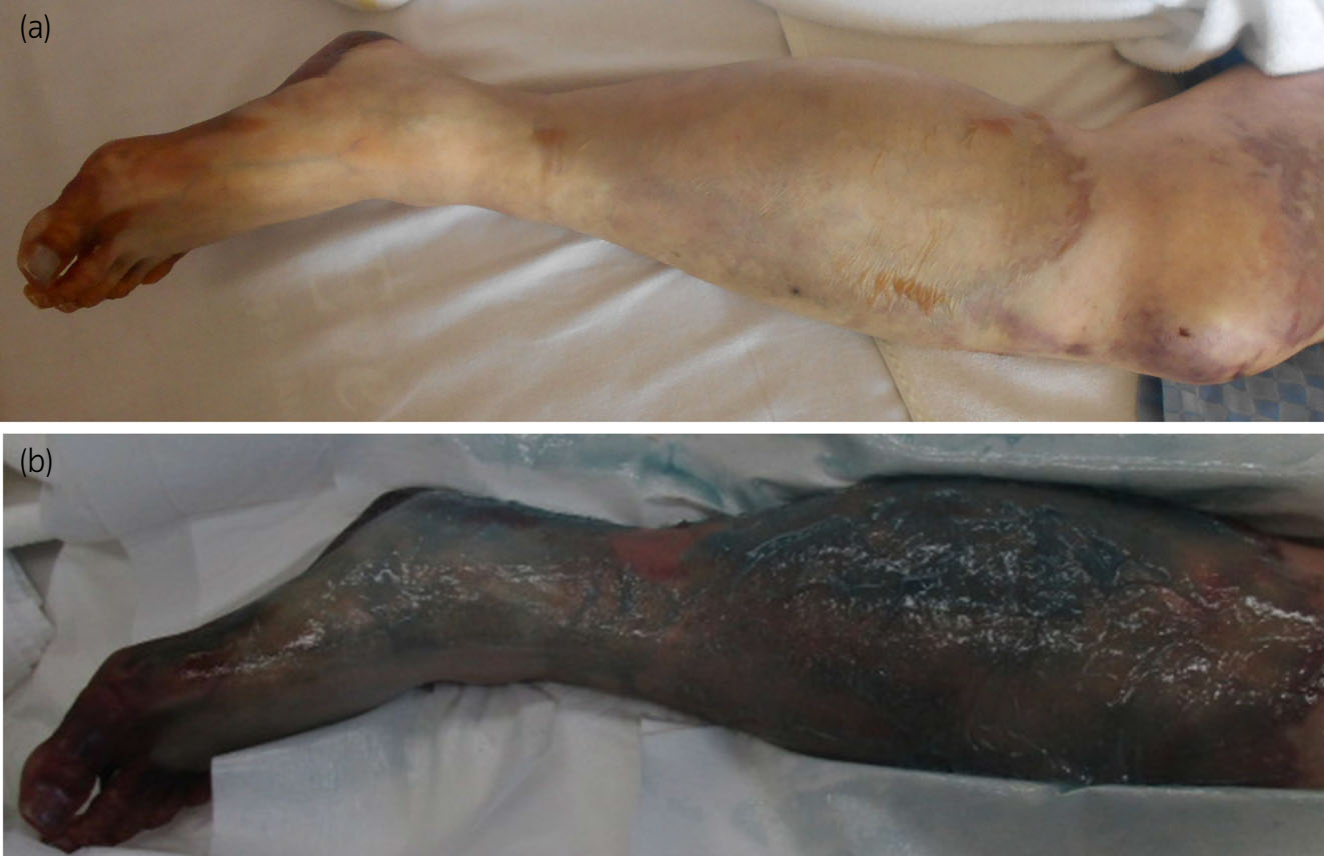
The amputated left leg. The left leg just before the sudden onset of leg pain (a) and just before amputation (covered by dimethyl isopropylazulene ointment) (b).

The patient was transferred to the general ward on Day 10. On Day 12, he developed sudden pain in the left leg, the color of which became purple (Fig. 3a). Ultrasonography showed the left ventricle without a tumor/thrombus and a thrombus from the common femoral artery to the popliteal artery, suggesting the thrombus in the heart caused thrombosis. CT confirmed the diagnosis. There have been multiple reported cases of pheochromocytoma resulting in arterial embolization.^7^ Excess catecholamines have been reported to contribute to hyper‐coagulability directly^8^, ^9^ and we considered that it resulted in femoral artery embolism from the intracardiac thrombus. We initially planned bilateral adrenalectomy as a cytoreduction surgery, which is reported to improve overall survival.^10^ However, we had to abandon the operative option due to the deterioration of the patient's general condition. Since left leg necrosis progressed without apparent infection, a half dose of chemotherapy was administered using cyclophosphamide, vincristine, and dacarbazine on Day 16 for disease control rather than amputating the leg. However, on Day 22, the left lower limb was severely infected and amputated (Fig. 3b). Consciousness disturbance persisted thereafter but gradually improved with the discontinuation of metyrosine on Day 40 (Fig. 2).

**Fig. 4:**
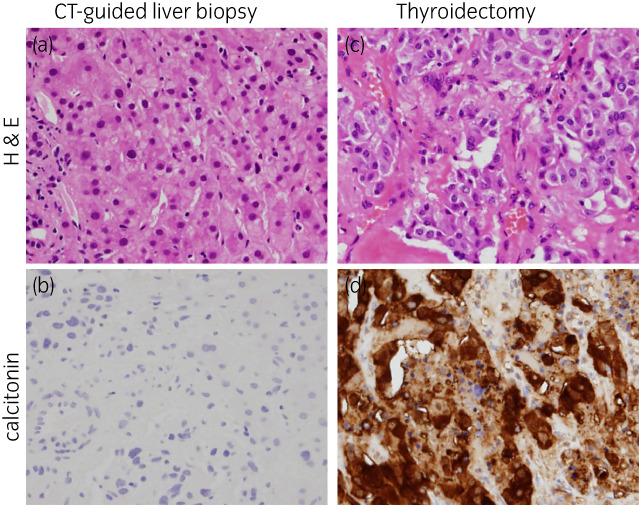
Pathology of the present case. (a,b) A pathological specimen of CT‐guided needle biopsy of liver metastases (L and l, hematoxylin and eosin [H&E] and calcitonin staining, respectively), negative for calcitonin, suggesting the metastasis of pheochromocytoma. (c,d) A pathological thyroid specimen after total thyroidectomy (M and m, H&E and calcitonin staining, respectively), positive for calcitonin, suggesting MTC. (a,c) hematoxylin and eosin staining. (b,d) immunohistochemistry for calcitonin.

On Day 80, the results of genetic testing revealed MEN2A with a pathogenic single nucleotide variant at codon 634 of the RET gene (NM_020975.6(RET):c.1900T>C). We screened for variants in *SDHB*, *SDHD*, *VHL*, *MAX*, and *RET* in a blood sample using the conventional direct Sanger sequencing method, as previously reported,^2^ after receiving written informed consent under our Institutional Review Board (approval #: 19–047). This result allowed us to perform the screening on the patient's children at an early stage. At first, we speculated that multiple metastases were from MTC rather than pheochromocytoma, because pheochromocytoma in MEN2A has been reported to be almost exclusively benign and localized to the adrenal.^11^ The elevated serum calcitonin level (130.0 [0.0–9.5] pg/mL) was consistent with this speculation. We were considering treatment with vandetanib, a *RET* inhibitor, if the liver metastasis was found to be of MTC origin. However, the liver biopsy revealed that metastasis originated from pheochromocytoma (Fig. 4a‐b). On Day 110, total thyroidectomy was performed, revealing calcitonin‐positive MTC with lymph node metastases (Fig. 4c‐d). The postoperative serum level of calcitonin was normalized (8.2 pg/mL), supporting MTC being localized to the neck (pT1bN1M0), which was consistent with moderately elevated calcitonin level.

The patient received two additional cycles of CVD therapy at 75 and 100% doses and underwent hepatic artery embolization to treat right liver metastases^7^; however, he died of disease progression on Day 221.

## Discussion

We encountered severely metastasized pheochromocytoma in a patient with MEN2A who exhibited various critical complications, including a thrombus and crisis due to excessive catecholamine. Among all cases of pheochromocytoma, the actual malignant potential is found in 2–13% of cases.^12^, ^13^, ^14^ Malignant potential is rarer in MEN2A cases,^6^ and to the best of our knowledge, there has been no case report of a patient with MEN2A dying of pheochromocytoma. This case may harbored MTC and pheochromocytoma from childhood, and the malignant potential of pheochromocytoma overtook that of MTC.

Based on genetic profiling, PPGL has recently been classified into cluster 1 or 2.^15^, ^16^ Cluster 1 represents germline or somatic gene mutations resulting in a dysfunctional hypoxic/pseudo‐hypoxic response with a central role for hypoxia‐inducible factor (HIF)1 α and HIF2 α, which are the main components of the response to low oxygen levels. In contrast, cluster 2 represents gene abnormalities in the activation of kinase signaling pathways, in which variants in the RET gene are included, as detected in the present case. The oncogenic RET has been shown to activate PI3K/AKT and RAS/RAF/MAPK dependent pathways, and its eventual activation of mTOR may constitute a common mechanism of tumor development.^17^ Based on molecular/genetic profiling, several clinical trials are currently underway.^18^ The development of molecular targeted therapy and/or immunotherapy, which has been widely used for other cancers, is expected for this disease, such as vandetanib.

Our case highlights the potential value of genetic testing and serum calcitonin measurement in diagnosing MEN2A. The diagnosis of MEN2A is relatively facile since genetic testing is covered by national insurance in Japan when MTC precedes pheochromocytoma, while its diagnosis is more elusive when only pheochromocytoma is initially evident. Measurement of serum calcitonin levels is readily available, and it may be feasible to measure it in patients with pheochromocytoma routinely.

## Author contributions

Koshiro Nishimoto: Project administration; supervision; validation; writing – original draft; writing – review and editing. Noriaki Santo: Data curation; project administration; resources; visualization; writing – original draft; writing – review and editing. Masato Yonamine: Resources; writing – review and editing. Kazuhiro Takekoshi: Formal analysis; investigation; supervision. Go Kaneko: Supervision. Suguru Shirotake: Supervision. Hisayo Fukushima: Investigation; methodology; supervision. Yoshitaka Okada: Formal analysis; supervision; validation. Masanori Yasuda: Resources; supervision. Akihiro Sakurai: Formal analysis; supervision. Masafumi Oyama: Supervision. Kent Kanao: Supervision.

## Conflict of interest

The authors declare no conflict of interest.

## Approval of the research protocol by an Institutional Reviewer Board

We screened for variants in *SDHB*, *SDHD*, *VHL*, *MAX*, and *RET* in a blood sample using the conventional direct Sanger sequencing method after receiving written informed consent under our Institutional Review Board in Saitama Medical University International Medical Center (approval #: 19–047).

## Informed consent

Yes.

## Registry and the Registration No. of the study/trial

None.
